# Anterior Cruciate Ligament Injury among Patients with Knee Injury Visiting the Out-patient Department of Orthopaedics of a Tertiary Care Centre: A Descriptive Cross-sectional Study

**DOI:** 10.31729/jnma.7402

**Published:** 2022-10-31

**Authors:** Sabin Pokharel, Sunil Singh Thapa, Arjun Prasad Lamichhane

**Affiliations:** 1 Department of Orthopaedic and Traumatology, Institute of Medicine, Maharajgunj, Kathmandu, Nepal

**Keywords:** *anterior cruciate ligament*, *diagnosis*, *history*, *orthopaedics*

## Abstract

**Introduction:**

Anterior cruciate ligament injury diagnoses are often missed at initial presentation. Though better diagnosed by physical examinations when done by orthopaedics surgeons, proper history is also important in diagnosing it. This study aimed to find the prevalence of anterior cruciate ligament injury among patients with knee injury visiting the out-patient Department of Orthopaedics of a tertiary care centre.

**Methods:**

This descriptive cross-sectional study was conducted on patients visiting the out-patient Department of Orthopaedics of a tertiary care centre within the study period from 31 January 2019 to 1 February 2020, after obtaining ethical approval from the Institutional Review Committee [Reference number: 321(6-11-E)2/075/076]. The anterior cruciate ligament injury diagnosis was made using a Magnetic Resonance Imaging scan. They were inquired about the specific history features at the time of injury: leg giving way, inability to continue the activity, massive swelling of knee joint within 6 hours, and 'pop' heard or felt. Point estimate and 95% Confidence Interval were calculated.

**Results:**

Among 127 cases of knee injury, anterior cruciate ligament injury was found in 109 (85.83%) (79.76-91.89, 95% Confidence Interval). History of the leg giving way, inability to continue the activity, massive swelling of the knee and 'pop' heard or felt were present in 90 (82.60%), 92 (84.40%), 91 (83.50%), and 86 (78.90%) cases of anterior cruciate ligament injury respectively. At least two history features were present in 104 (95.41%) cases.

**Conclusions:**

The prevalence of anterior cruciate ligament injury was found to be similar to the published studies.

## INTRODUCTION

An anterior cruciate ligament (ACL) is commonly injured but underdiagnosed.^1^ The physical examination for ACL is accurate when performed by an experienced Orthopaedic Surgeon when compared to a physician.^2^ The diagnosis of acute ACL injury can be made using history features present at the time of trauma.^3^ Some studies suggest typical history features in ACL tear at the time of the trauma but are inconsistent.^3-5^

ACL injury is a global problem, and its incidence is rising.^3^ Use of history features can be done by all physicians in the diagnosis of ACL injury and would reduce the delay in diagnosis and the complications and would result in prompt referral to a specialist.^3^

So, this study aimed to find out the prevalence of anterior cruciate ligament injury among patients with knee injury visiting the outpatient department of orthopaedics of a tertiary care centre.

## METHODS

This descriptive cross-sectional study was done in a tertiary care centre from 31 January 2019 to 1 February 2020, after the ethical approval from the Institutional Review Committee of the Institute of Medicine [Reference number: 321(6-11-E)^2^/075/076]. The cases of knee injury who presented to the Out-patient Department (OPD) of Orthopaedics of Tribhuvan University Teaching Hospital (TUTH) from the age of 16 years to 60 years were included in this study. Cases with a multi-ligamentous knee injury, current fracture around the knee joint, prior history of ACL reconstruction in the same knee, patients not giving consent, and patients with contraindicating features for Magnetic Resonance Imaging (MRI) study were excluded from the study. A convenience sampling technique was used. The sample size was calculated using the following formula:


$$\begin{array}{ll} \text{n} & ={\text{Z}}^{2}\times \frac{\text{p}\times \text{q}}{{\text{e}}^{\text{2}}}\\ & =1.96^{2}\times \frac{0.86\times 0.14}{0.07^{2}}\\ & =95 \end{array}$$

Where,

n= minimum required sample size
Z= 1.96 at 95% Confidence Interval (CI)
p= past prevalence of anterior cruciate ligament injury, 86%^6^
q= 1-p
e= margin of error, 7%

The minimum required sample size was 97. However, a total of 127 samples were taken. Written consent was obtained, and specific questions regarding the history features present at the time of trauma were inquired. The patients were screened for ACL injury with Anterior Drawer Test and Lachman test, and if signs of laxity were present, then the patients were subjected to an MRI scan for confirmation of ACL injury.^7,8^ The history features inquired were feeling of "leg giving way" (knee going out of place), "inability to continue the activity", "massive swelling around knee joint within 6 hours of trauma", and "hearing or feeling of 'pop' sound". The presence of a particular history feature was taken as 'yes'; when not present, it was taken as 'no', and if the patient is not sure about the presence of that feature, it was taken as 'not sure' or 'not applicable' which is the LIMP index.^3^ Demographic details of the patient and the date of trauma as recalled by the patient were noted. The total days from the date of trauma to MRI diagnosis were taken as delays in diagnosis.^3^

Data analysis was done using IBM SPSS Statistics 26.0. Point estimate and 95% CI were calculated.

## RESULTS

Out of 127 knee injury cases, anterior cruciate ligament injury was seen in 109 (85.83%) (79.76-91.89, 95% CI) cases. A total of 104 (95.41%) cases had at least two of the studied history features present at the time of trauma. Non-contact mode of injury was seen in 67 (61.47%), and most were due to non-sporting activities in 64 (58.72%). The injury of ACL was accompanied by medial meniscus (MM) tear in 41 (37.61%) cases, and ACL combined with lateral meniscus (LM) injury in 13 (11.93%) cases (Table 1).

**Table 1:** Demographic details of patients and injury characteristics (n= 109).

**Injury characteristics**		n (%)
Limb involved	Right	56 (51.38)
	Left	53 (48.62)
Mode of injury	Non-contact	67 (61.47)
	Contact	42 (38.53)
Activity at time of injury	Non-sporting	64 (58.72)
	Football	22 (20.18)
	Futsal	15 (13.76)
	Basketball	4 (3.67)
	Volleyball	2 (1.83)
	Other sports	2 (1.83)
MRI diagnosis	ACL injury	41 (37.61)
	ACL+MM injury	41 (37.61)
	ACL+MM+LM injury	14 (12.84)
	ACL+LM injury	13 (11.93)

The mean age was 30.28±9.65 years. In this study, there were 31 (28.44%) females and 78 (71.56%) males. Among females, only 1 (3.22%) case accounted for sporting activity as the cause of the injury and the rest of all 30 (96.77%) cases were due to non-sporting activities.

Among the history features reported by cases at the time of injury, "inability to continue the activity" accounted for the most reported features as seen in 92 (84.40%). 'Pop' heard or felt was the least reported feature in 86 (78.89%) cases (Table 2).

**Table 2:** History features present at the time of trauma leading to anterior cruciate ligament injury (n= 109).

History features present at the time of trauma	Response	n (%)
Leg giving way	Yes	90 (82.57)
	No	16 (14.68)
	Not sure	3 (2.75)
Inability to continue the activity	Yes	92 (84.40)
	No	17 (15.60)
	Not applicable	-
Massive swelling of the knee within 6 hours of trauma	Yes	91 (83.49)
	No	18 (16.51)
	Not sure	-
'Pop' heard or felt	Yes	86 (78.90)
	No	19 (17.43)
	Not sure	4 (3.67)

The majority of cases, 57 (52.29%), reported all the four history features present at the time of trauma. About 5 (4.59%) cases reported at least one feature present among the possible four history features. The cumulative percentage of cases reporting two or more history features present was 104 (95.41%) (Table 3).

**Table 3:** Numbers of history features present and the cumulative percentage (n= 109).

Numbers of 'LIMP Index' history features	n (%)	Cumulative Percentage
4	57 (52.29)	52.29
3	31 (28.44)	80.73
2	16 (14.68)	95.41
1	5 (4.59)	100

Delay to diagnosis ranged from 6 to 3743 days (nearly 10 years). The mean delay to diagnosis was 343.33±670.39 days.

## DISCUSSION

In our study, the prevalence of anterior cruciate ligament injury was found in 85.82%. This study found that 95.41% of cases of ACL injury had more than two of the studied history features present at the time of trauma. ACL injuries are attended to first at the Emergency Department, where an Orthopaedic Surgeon may not be available for physical examinations. Hence, the cases are underdiagnosed or wrongly diagnosed as sprains. Using the history features alone, an Emergency physician can screen the case for ACL injury and timely referral for further evaluation to an orthopaedic surgeon, thus reducing the delay to diagnosis and the subsequent complications.

The prevalence of ACL injury was found to be similar in the present study to the study done in the Indian city of Chandigarh.^6^ In the rural Indian population having a similar lifestyle as the present study population, the prevalence was found to be 70% which was a bit less than the present study.^8^ It may be because, in the retrospective part of the study, patients included were only the ones that were admitted, and patients in the out-patient department may have been missed.

In the present study, non-sporting activity was the cause of ACL injury in 64 (58.7%) cases. This finding was contrary to many studies suggesting sporting activity as the commonest activity at the time of injury.^1,2,5,9^ This could be because of the fact that the population under the present study are less likely to adopt sports as a recreational activity. The similarity was seen in a study done in 2019 in the rural Indian population, which shares a similar population to the present study.^8^

The use of different history features in different numbers^1,5,10,11^ in many studies shows that consistent history features pertaining to ACL injury have not yet been defined reliably. A multisite prospective crosssectional study done in the United Kingdom used the survey questionnaire in different NHS Hospital Trusts locatedwithin the West Yorkshire and North Lincolnshire regions to recruit 194 cases of ACL injury.^3^ Four history features at the time of trauma were studied like the present study and abbreviated as the 'LIMP index'. When a threshold of two or more features out of four was used, 186 (95.87%) cases had been diagnosed correctly. Similar findings, 104 (95.41%) were found in the present study. The majority of cases 57 (52.29%) in the present study also reported the presence of all four history features of 'LIMP index'. In the present study, findings of four history features were fairly comparable to this study as well.

The feeling of subjective improvement and return of the ability to bear weight on the injured knee occurring after 1-2 weeks as described in another study done in the United Kingdom, has not been considered in the history features in the present study as these are seen much later after trauma episodes.^5^ Rather, the present study studies history features present acutely at the time of trauma. The pop sound was reported in very few cases as compared to the present study. This can be because the present study has considered hearing and feeling pop both in contrast to the above study, which only considers hearing pop as a present.

In the study done at the University of Cincinnati Sports Medicine Institute, the United States, different history features were inquired about retrospectively in 103 patients.^12^ Knee giving way, unable to continue, knee swelling, and pop sound heard were among the inquired history features through a questionnaire. Other history features were fairly comparable to the present study, but the pop sound was seen only in 65% of cases which is less than the present study. This study also had not taken the feeling of pop rather, only hearing has been accounted so less number may have been reported.

A prospective multicenter diagnostic study done in the Canadian population and another cross-sectional descriptive study done in Netherlands included physical examinations and history features in combination for the diagnosis of complete ACL tears.^11,13^ The negative physical findings with negative history features were able to exclude ACL injury. But in the present study, only history features were analysed without the use of physical examination.

This study explores the prevalence of ACL injury, the presence of different history features. However, there is a need to further look for the sensitivity and specificity of using these history features for the diagnosis of ACL injury. Recall bias on the side of a patient could not be ruled out.

## CONCLUSIONS

The prevalence of anterior cruciate ligament injury was found to be similar to the reported literature. All four history features; leg giving way, inability to continue the activity, massive swelling of the knee within 6 hours, 'pop' heard or felt, were present in most cases of the anterior cruciate ligament injury, which is similar to findings of the previous studies. But, further exploration of the sensitivity and specificity of history features needs to be done.
